# Editorial: Global excellence in nephrology: Asia and Australasia

**DOI:** 10.3389/fneph.2023.1248728

**Published:** 2023-07-14

**Authors:** Cheng Xue, Mingxi Li, Suxia Wang

**Affiliations:** ^1^ Division of Nephrology, Shanghai Changzheng Hospital, Second Military Medical University (Naval Medical University), Shanghai, China; ^2^ Department of Nephrology, Peking Union Medical College Hospital Chinese Academy of Medical Sciences (CAMS), Beijing, China; ^3^ Department of Nephrology, First Hospital of Peking University, Beijing, China

**Keywords:** IgA nephropathy, minimal change disease, polycystic kidney disease, chronic kidney disease, end-stage kidney disease

Global collaboration is the cornerstone of current scientific advancement in nephrology. Within the realm of nephrology, the regions of Asia and Australasia have emerged as hotbeds of innovation, research, and clinical excellence. With a rich diversity of patient populations, unique healthcare challenges, and a strong commitment to advancing renal care, Asia and Australasia have become recognized for their contributions to global excellence in nephrology.

In recent years, the field of nephrology in Asia and Australasia has undergone a remarkable transformation, leading to groundbreaking discoveries, improved patient outcomes, and a positive impact on public health. These achievements have been the result of concerted efforts by healthcare providers, researchers, and allied professionals who have strived to tackle the challenges posed by kidney diseases in the region.

One of the key drivers of excellence in nephrology in Asia and Australasia has been the advancement of research and clinical practice. Researchers and clinicians in the region have made significant contributions to various aspects of nephrology, including hereditary nephropathy, renal pathology, glomerulonephritis classification, prognosis, and the development of new treatments. These advancements have not only deepened our understanding of kidney diseases but have also translated into tangible improvements in patient care, diagnosis, and treatment options.

In this Research Topic, the study by Guo et al. investigated the clinical and pathological characteristics of patients with IgA nephropathy (IgAN) with and without associated minimal change disease (MCD). The study found that patients with MCD-IgAN had lower levels of serum galactose-deficient immunoglobulin A1 (Gd-IgA1) than those with non-MCD-IgAN but presented with higher levels than healthy participants. In comparison to non-MCD-IgAN, complement C3 activation in the circulation and the renal location was substantially weaker in MCD-IgAN. The authors suggest that IgAN with MCD might be MCD with coincidental IgA deposition. This study provided valuable insights into the clinical and pathological characteristics of patients with IgAN with and without MCD.

In a second study, Liu et al. investigated the relationship between serum phosphate levels and mortality in 23,283 septic patients. The study found that both hypo- and hyperphosphatemia were independent risk factors for mortality, and that dynamic changes in phosphate levels were also associated with increased mortality risk. There is a U-shaped association between serum phosphate levels and the risk of mortality in septic patients.

A review by Zhang et al. provided an overview of the potential therapeutic effects of plant-derived compounds in treating autosomal dominant polycystic kidney disease (ADPKD), which is the leading hereditary kidney disease. The article highlighted the limitations of current drugs used to treat ADPKD and suggested that plant-derived compounds may offer a promising alternative. Plant-derived compounds, including saikosaponin-d, Ganoderma triterpenes, curcumin, ginkgolide B, steviol, resveratrol, Sparganum stoloniferum Buch.-Ham, Cordyceps sinensis, triptolide, quercitrin, naringin, cardamonin, gambogic acid, and olive leaf extract, have shown potential in delaying the development of cysts or improving renal function in ADPKD. These plant-derived compounds act on various signaling pathways involved in the pathogenesis of ADPKD, such as the RAS/MAPK pathway and intracellular calcium levels, and may serve as promising candidates for future therapeutic interventions.

Despite the impressive strides made in nephrology, Asia and Australasia continue to face unique challenges in managing kidney diseases. The prevalence of chronic kidney disease (CKD) and end-stage kidney disease (ESKD) remains high in these regions, driven by factors such as an aging population, increasing rates of diabetes and hypertension, and lifestyle changes. Limited healthcare resources, inadequate infrastructure, and disparities in access to specialized care pose significant hurdles to effective kidney disease management. The study by Nagai et al. discussed the relationship between urban design and CKD. The prevalence of CKD has increased in Japan due to the aging population, and lack of physical activity is a major preventable risk factor for mortality in adults. Rural city planning in Japan can improve renal health outcomes by promoting walking, bicycling, and the use of public transportation, which can increase daily physical activity levels and access to specialized medical care. Moreover, multidisciplinary education without medication for obese populations can positively impact obesity through increased physical activity, and recreational walking is a dominant contributor to increasing step counts.

To further overcome these challenges and enhance the level of care, collaborative approaches have emerged as vital components of achieving global excellence in nephrology. Regional alliances, research consortia, and professional networks have facilitated the sharing of knowledge, expertise, and best practices among nephrology communities in Asia and Australasia. By fostering multidisciplinary collaborations between clinicians, researchers, epidemiologists, geneticists, and allied healthcare professionals, these initiatives have catalyzed innovation, accelerated research progress, and promoted the development of region-specific policies and guidelines.

In summary, the region of Asia and Australasia has emerged as a powerhouse of global excellence in nephrology, demonstrating remarkable advancements, overcoming challenges, and fostering collaborative approaches to enhance patient care. By leveraging research and addressing the evolving burden of kidney diseases, Asia and Australasia will continue to make significant contributions on a global scale to the field of nephrology and improve the lives of individuals affected by kidney diseases.

## Author contributions

All authors listed have made a substantial, direct, and intellectual contribution to the work and approved it for publication.

